# Medical and Physical Intelligence

**Published:** 1823-06

**Authors:** 


### MEDICAL AND PHYSICAL INTELLIGENCE.

### ANATOMY.

1. *Unusual Multiplication of Muscles.*—DR. FR. TIEDMANN relates that, when opening the body of a young and very muscular man, who appeared to have been very athletic, he met with a second pectoralis major muscle under the ordinary one,—also a double pectoralis minor; and, on examining this subject still further, he found two glutei majores and two circulares, on each side of the body.—*(Med. Chirurg. Zeitung.)*

2. *Double Uterus and Vagina.*—Dr. TIEDMANN communicates a very remarkable case of the above description, the preparation of which is to be found in the Museum of Heidelberg. In this individual the vagina and uterus were found both to be double, the latter of which was distended from impregnation. The left uterus was in the same condition as it usually is after delivery, the person from whom the preparation was taken having died on the nineteenth day after parturition: the right, on the other hand, had all the appearances which those who never have borne children usually possess.—In the same communication, the author alludes to two similar cases, which he had himself met with on former occasions.—*(Med. Chirurg. Zeitung.)*

3. *Œsophagus wanting.*—The subject of this singular case was a child, met with by Dr. SONDERLAND, of Bremen. A few hours after birth, a little sugar and water was given to the infant, as is usually done in similar circumstances: it swallowed the fluid with avidity, but immediately afterwards it was rejected by the nose and mouth; the infant at the same time being convulsed, affected with a hoarseness, and becoming blue in the face. It died on the eighth day, from actual starvation. On examination after death, the pharynx was found to terminate as usual in the œsophagus; which, however, about opposite to the third vertebra of the neck, became quite impervious, and there terminated, without forming any sac or other deformity. From this point to the stomach, not the slightest vestige of an œsophagus was met with: it was entirely wanting.—*(Med. Chirurg. Zeitung.)*

### PHYSIOLOGY.

4. *Motions of the Eye.*—A paper, in illustration of the uses of the muscles connected with the eye-ball, was lately communicated to the Royal Society, by Mr. CHARLES BELL. The object of these inquiries is to show that certain motions are performed by the eye, which have not hitherto been described. Thus, every time the eye-lids are brought together to cover the globe, the eye turns upwards; without which motion it would not be properly moistened, nor the particles of dust removed from its surface. Next it is shown that during sleep the ball is turned up, so as to lodge under the superior palpebra. These movements are rapid and involuntary, while others are under the government of the will, and for the purpose of directing the eye to different objects. Both the oblique and straight muscles have been regarded as voluntary; but Mr. Bell maintains that the oblique are for the performance of the insensible motions, and the recti muscles for those under the command of volition. According to this view, we are enabled to judge of the distance and relative position of objects by the consciousness we have of the action of the straight muscles. A further inference is, that the functions of these muscles are connected inseparably with the state of the retina, being active only so long as the sense of vision is in activity: when this ceases to act, the oblique muscles come into play, and draw the pupil upwards beneath the upper eye-lid. Hence that turning of the eye-ball which we witness in sleep, in fainting, or in death, is but the indication of insensibility. In another paper the author purposes to explain the uses of the numerous nerves going to the orbit, on the principles of the foregoing remarks.

5. *Irritability of the Tongue.*—The following account is from the pen of BLUMENBACH:

I had the tongue of a four-year-old ox, which had been killed in the common way, by opening the large vessels of the neck, cut out in my presence, while yet warm, and at the same time the heart, in order that I might compare the oscillatory motion of this organ (which is by far the most irritable that we are acquainted with,) with the motion of the tongue. And, when I excited both viscera at the same time by the same mechanical stimuli,—namely, incisions of a knife and pricks of a needle,—the divided tongue appeared to all the bystanders to survive the heart by more than seven minutes, and to retain the oscillations of its fibres altogether for a quarter of an hour; and so vivid were the movements when I cut across the fore-part of the tongue, that the butcher's wife compared them to those of an eel in a similar condition, quite in the way that Ovid has compared them to the motions of the tail of a mutilated snake.

To these observations made upon animals, I may add here a similar one made upon the human tongue itself, the knowledge of which I owe to my excellent friend and much-respected colleague, Reimar.

A boy in Hamburgh, who was severely affected with epilepsy, bit the forepart of his tongue in a violent paroxysm, in such a manner that it adhered only by a thin slip. This segment, therefore, as being not only useless but very inconvenient to the patient, it was immediately judged necessary to cut off: and when the physician, the illustrious Chaupefié, placed it upon his hand, he was surprised to see it palpitate strongly. In order, however, to guard against all deception, as the motion might have depended upon the action of the muscles of his hand, he placed the bit of tongue in the bottom of a window, and even then it continued to move for several minutes, insomuch that it even seemed, as all the bystanders testified with one accord, to change place a little, and creep forward. External stimuli, the prick of a needle, or the application of salt, also excited similar motion; scarcely, however, differing in any way from the spontaneous ones.

I am therefore much mistaken if the muscular substance of the tongue is not possessed of a remarkable degree of irritability; and Ovid has described its phenomena with exquisite precision.—(*Edinburgh Philosophical Journal.*)

---

PATHOLOGY.

6. *On the Xanthoöpia of Jaundiced Persons.*—The rarer xanthoöpia, as it may be called, of jaundiced persons, (says M. Blumenbach,) seems to be in this condition, that it supposes at first a yellowish tinge in the pellucid media which transmit the rays of light, especially of the aqueous humour and crystalline lens; then a not so gentle, but more sudden, attack or increase of the disease; and, lastly, both a vivid perception in the sensorium and application of the mind. But, as these seldom happen at once in icterus, we can easily comprehend why very few patients in that disease complain of the yellow tint of objects. On the contrary, I knew a lady of excellent talents and accomplishments, who, on a sudden and severe attack of icterus, saw at first linen of all kinds, towels, &c. of a livid or dusky hue, and as if they had been ill washed, or not properly bleached. But those upon whom the disease seizes slowly, advancing as it were step by step, no more experience this yellow hue of objects than old people do, in whom we have known the crystalline lens become livid in very advanced age. Should this tinge take place in one eye only, the other remaining uncontaminated, that diversity of colour in white and shining objects would be no less easy of observation, than in patients who, after depression of cataract, see the objects, from the use of sulphate of copper applied to the diseased eye, on which they look with it, of a colour sometimes verging towards blue. And a somewhat similar vitiation of sight is known to take place in those who have for a long time kept one eye applied to dioptrical instruments. Thus the celebrated philosopher James Rohault, after looking, without interruption, for twelve hours at a distant battle, by means of a telescope, from that time forth saw every object with his right eye, which he had here fatigued so much, of a different colour from what it had when viewed with the left. And the same circumstance happened to myself, after being assiduously employed for several days, in making observations with a compound microscope.—(*Edinb. Phil. Journal.*)

7. *Case of Boulimia.*—Dr. Crane was called to a lady, aged twenty-six years, who was affected with an extraordinary appetite, so as to consume at each repast three or four pounds of meat, without reckoning bread and other viands. This state affected the lady so much, that she would never consent to eat in the presence of a stranger, or even of her own family. In general she was obliged to vomit a short time after her meals, when she rejected what she had swallowed, accompanied with viscid matter, resembling the white of egg, and having a sour taste. Shortly after, she took a large quantity of food again, which she vomited in a similar manner. Several physicians were now consulted, who attributed the complaint to disease of the pylorus. A great variety of medicines were employed without success, when all at once the lady was seized with continued fever, one of the leading symptoms of which was loss of appetite, but which returned with the same intensity as soon as the fever subsided. This loss of appetite during the fever, and other circumstances, induced Dr. Crane to suppose that the boulimia arose from a particular irritability of the stomach, increased by the presence of aliments; in consequence of which he resolved to give his patient nothing but liquids, such as milk, sago, arrow-root, &c. No relief followed this measure; and Dr. C. confined the diet to soup and nutritive glysters. At the end of six weeks, soup with bread was allowed, and the solid food gradually increased; the appetite then became natural, and has continued so ever since, a period of nine years.—Some pills were given in this case, but their composition is not mentioned.—(*Journal der Practischen Heilkunde, von* W. HUFELAND.)

8. *Partial Paralysis of the Retina.*—A furrier, aged sixty, was attacked with a violent pneumonia, attended with great difficulty of breathing and cerebral congestion. He was bled several times; rigid diet, leeches, and pectoral drinks, were prescribed. The patient became convalescent: he had neither suffered confusion of the head nor noises in the ears, when on a sudden he felt a violent pain in the right coronal region. This pain soon ceased; the patient went to sleep, and, on awaking, he found that his left eye was become the seat of a singular affection, which subsists at this time. The right eye being shut, all the objects that are placed on the outside of the axis of vision are invisible. He distinguishes perfectly those situated on the side of the nose. A sheet of white paper, placed before the left eye, appears cut into two parts,—the internal half visible, the other not. A black line traced upon a white ground, a stick held horizontally, afford the same result. If a person places himself altogether on his left side, and passes before him, he sees absolutely nothing: in short, he only sees the image when placed within the axis of the eye. The sight is very good in that part of the retina which still remains sensible; the iris is perfectly contractile in its whole extent; the pupil is rather larger than usual. The patient refused to submit to any treatment.—(*Bulletin de la Societé d'Emulation Medicale.*)

9. *Tumors of the Axilla.*—M. DUPUYTREN presented the Royal Academy of Medicine, at Paris, with the case of a young woman who had two tumors, one in the right axilla, and a second, somewhat smaller, behind the shoulder of the same side. The anomalous character of these swellings forbad an operation; but the patient, fearing this result, quitted the Hotel Dieu. But the growth of these tumors, and evident symptoms of an affection of the heart and the large vessels, compelled her to return to the hospital. Some time after this she died. On dissection, it was found that the swelling behind the shoulder communicated with that under the axilla, and this was extended by a sort of pedicle between the clavicle and first rib, penetrated into the chest, and was lodged in the superior vena cava. This vessel was filled to a great extent by the accumulation of the cancerous matter which composed the bulk of these tumors.—Is it probable that this organic alteration, developed in the vena cava, had extended itself so as successively to form the two swellings? Every thing leads one to suppose that this is the order in which the pathologic phenomena succeeded each other.—(*Revue Medicale*, Fevrier.)

THERAPEUTICS.

10. *Vinegar in Colica Pictonum.*—Dr. KOLMODIN, in the southern district of Gothland, has related a case of colica pictonum, which he met with in a painter of that country. After trying a great many different remedies, some of which are said to be often useful, without the slightest benefit whatever, he at last administered frequent doses of wine-vinegar, in conjunction with Epsom salts: when the medicine had freely opened the bowels, every symptom left the patient, and next day he was quite free from complaint.—(*Svenska Läkare Sällskapeto Handlingar, Sjunde Bandet.*)

11. *Waters containing Carbonic Acid.*—It results from many observations made by M. DESPORTES, that the use of Seltzer water, either natural or artificial, the water of Pougues, or, generally speaking, all those waters containing carbonic acid gas, should not be lightly recommended, and that the idiosincracy of patients should previously be consulted; and that the first effects of such waters prescribed during meals, mixed with wine, should especially be observed. The author has seen the action of these waters derange the digestive functions, and, by sympathy, produce an irritation of the brain, which put on at first the character of intoxication: with several patients, severe nervous symptoms and a threatening of asphyxia were the consequences, and particularly violent pains in the region of the heart.—(*Revue Med.*)

SURGERY.

12. *Extraction of Stones from the Bladder.*—M. CAMPANA, considering urinary calculi in relation only to their shape, divides them into three classes,—spherical, flat or irregular, and oval. The first have only one diameter; the second have two, a great and a small diameter; whilst the third class has three,—a great, a middle, and a small one. In the first case, they can only be laid hold of by the forceps in one way; in the second class, he recommends them to be laid hold of by their large diameter; and the third class, by the middle one. If lithotomists have often varied their method of opening the bladder, says M. Campana, this has not been the case with respect to the removal of the stone; for all operators, to the present day, have agreed that the stone should be laid hold of by its smallest diameter: nevertheless, when taken hold of in this manner, it presents inconveniences which are not met with when it is taken hold of by the large diameter. The first of these inconveniences is, that, the large diameter being out of the forceps, the stone touches and wounds with its asperities the internal membrane of the bladder, which does not happen if the case be reversed; for then the smooth surface of the blades of the forceps facilitates the dilatation of the opening, without offending the parts. The second inconvenience is still greater, and is produced by the angles formed by the grasping portion of the forceps, which are applied directly to the membranous circle of the sphincter of the bladder, and which makes the extraction of the stone impossible; or, at least, renders the tearing of the neck of the bladder inevitable, the consequences of which are fatal. The difficulties and danger are increased by the contraction of the bladder, stimulated by the contact of the forceps and the calculus; and it is easily conceived what mischief may ensue in extracting the stone under such circumstances. All this is avoided by laying hold of the stone by its greatest diameter. It is true that by this method the forceps is much more open, which requires that the opening into the bladder should be more dilated; but this dilatation, says M. Campana, is readily obtained when the bladder contracts not upon the stone, nor upon the angles of the forceps, but merely upon their smooth back. They then act only as simple dilators, in a similar way to the forceps in the extraction of the fœtus. M. Campana proves the superiority of this plan by the operations he has himself performed, and relates the case of a youth, aged sixteen, who was cured in twenty-nine days without the slightest accident, from whose bladder he extracted a stone, the large diameter of which was two inches.—(*Annali Univ.*)

### PHARMACY.

13. *New Method of Preparing Nitric Ether, by* M. DUROZIER.—Struck with the difficulty of succeeding by the methods in use, and, above all, with the inconvenience resulting from the application of heat in this preparation, M. Durozier conceived that, in developing the degree of heat requisite for the re-action of the acid on the alcohol, by their simple admixture, this process might be attended with more certainty. Sulphuric acid appeared to him most proper for this purpose. Again, wishing to get rid of the complicated apparatus of Wolfe, he employed the common means of distillation which are to be found in every laboratory. He placed a tubulated retort, containing about six pints, in a sand bath; the neck entered directly into a serpentine tube, to the other end of which was adapted a receiver, placed in a vessel fitted to keep it cool. At the superior part of the receiver was a safety-tube, communicating with a flask containing a little alcohol, for the purpose of absorbing any ether which might escape. The apparatus being thus arranged, he took three pounds of alcohol at 36°, and mixed with it one pound eight ounces of nitric acid at 32°. (This mixture was made the evening before.) The whole was introduced into the retort, and immediately after he poured twelve ounces of concentrated sulphuric acid upon it: the tube was adapted and secured with lute. Five minutes after the introduction of the sulphuric acid, ebullition became manifest; streaks of ether marked the sides of the retort; and soon after it flowed abundantly from the inferior extremity of the tube. When the ebullition had ceased, he removed the contents of the receiver, and found it to weigh twenty-three ounces; he then agitated it with an equal quantity of water, and, after standing for a moment, the ether floated pure and limpid at the top. When separated, it weighed ten ounces three drachms. After this first result, the receiver was again placed, and a very moderate heat applied to the sand bath, so as to distil "a very pleasant and soft nitrous liquor."

On this process a report is made by MM. FAGUER and PETROZ, who repeated the experiment with much care, and who are assured that by this means "may be obtained, very quickly and with great facility, a much more considerable quantity of nitric ether than by any other known." They object, however, to the method of purification recommended by M. Duroziez, as insufficient, and advise that already generally employed.—(*Journal de Pharmacie*, Avril.)

---

MISCELLANEOUS.

14. *Annual Report from the National Vaccine Establishment, to the Right Honourable* ROBERT PEEL, *principal Secretary of State for the Home Department.*

*Percy-street; 21st March*, 1823.

SIR,—The more than usually numerous applications which have been made to us for vaccine lymph in the course of the last year, give us reason to believe that the confidence of the world in vaccination is increasing; and afford, at the same time, a strong proof of the wisdom and benevolence of Parliament in having provided an establishment where this resource is always to be procured. We have dispatched it, since our last Report, to the East and West Indies, to Ceylon, to the Cape of Good Hope, the island of Mauritius, to the coast of Africa, to New South Wales, &c. &c. We have also sent the lymph to France and to Italy, and to almost every part of this kingdom, and have received the cordial acknowledgments of our correspondents for the promptitude with which their sudden demands have been supplied. That this has been the means of preventing much calamity, we are well convinced; for the small-pox has prevailed as an epidemic with more than ordinary malignity in various parts of this island lately, and has committed great ravages in those districts where it found victims unprotected against it by a previous process. The advantages of vaccination in places subjected to these severe visitations, have been confessedly decisive and remarkable; those who had used this resource being observed to remain generally unhurt in the midst of danger; and, if there were any whom the contagion was able to infect, these were remarked, almost universally, to have the disease in that mitigated form which is not attended with danger.

We are not surprised, therefore, to find that, amongst the higher orders of society who think for themselves, and take the trouble of comparing probabilities and of drawing just inferences, vaccination has been used to a greater extent in the last than in former years. But, amongst the lower orders of the people, deep-rooted prejudices are difficult to be overcome; their improvidence renders them indifferent to advantages which are not of obvious and instant application; and terror alone, under circumstances of pressing and immediate danger, induces them to adopt this safe and easy course. These, therefore, it is the constant object and care of the Board to excite to a proper sense of the humane solicitude of the legislature for their safety, and to impress upon them the necessity of employing vaccination forthwith for their protection.

These warnings are more especially called for in those districts where the small-pox, by not having appeared lately, has lost something of its terrors; where the inhabitants have been betrayed thereby into a dangerous supineness, and the practice of vaccination has been neglected.

(*Signed by* Sir HENRY HALFORD, *and the other Members of the Board.*)

### METEOROLOGICAL JOURNAL,

*From April 20, to May 19, 1823.*

By Messrs. WILLIAM HARRIS and Co. 50, Holborn, London.

| | MOON. | Rain gauge | THERM. | | | BAROM. | | DE LUC'S HYG. | | WIND. | | ATMOSPHERIC VARIATION. | | |
|---|---|---|---|---|---|---|---|---|---|---|---|---|---|---|
| | | | 9 A.M. | MAX. | MIN. | 9 A.M. | 10P.M. | 9 A.M. | 10P.M. | 9 A.M. | 10P.M. | 9 A.M. | 2 P.M. | 10P.M. |
| Apr. | | | | | | | | | | | | | | |
| 20 | | | 42 | 53 | 40 | 29.72 | 29.84 | 65 | 69 | NNW | NW | Fine | | |
| 21 | | | 45 | 51 | 38 | 29.73 | 29.55 | 63 | 65 | WNW | ESE | Fine | | Fair |
| 22 | | | 41 | 49 | 41 | 29.50 | 29.43 | 64 | 70 | E | E var. | Fine | | |
| 23 | | .30 | 43 | 53 | 41 | 29.10 | 29.14 | 72 | 79 | SSE | ESE | Fair | Sho'ry | Rain |
| 24 | | .12 | 43 | 50 | 40 | 29.32 | 29.48 | 85 | 68 | NE | N var. | Clond. | Sho'ry | Clond. |
| 25 | ○ | | 42 | 51 | 39 | 29.55 | 29.63 | 69 | 75 | SW | WSW | Fine | | Sho'ry |
| 26 | | .49 | 41 | 48 | 37 | 29.49 | 29.55 | 90 | 100 | E var. | E var. | Rain | | |
| 27 | | | 41 | 50 | 38 | 29.33 | 29.98 | 77 | 72 | E var. | E var. | Fine | | |
| 28 | | | 44 | 52 | 40 | 29.97 | 29.92 | 73 | 71 | E var. | WSW | Fair | Fine | Fair |
| 29 | | | 45 | 50 | 43 | 30:04 | 30.18 | 74 | 66 | NNE | ESE | Fine | | |
| 30 | | .04 | 45 | 55 | 42 | 30.23 | 30.21 | 72 | 69 | ESE | W | Foggy | Fine | |
| May | | | | | | | | | | | | | | |
| 1 | | | 45 | 57 | 48 | 30.11 | 30.13 | 67 | 72 | NE | E | Foggy | Fine | |
| 2 | | | 52 | 62 | 49 | 30.16 | 30.10 | 67 | 70 | E | ESE | Fine | | |
| 3 | ☽ | | 55 | 65 | 48 | 30.11 | 30.12 | 68 | 71 | E | E | Fine | | |
| 4 | | | 52 | 54 | 49 | 30.20 | 30.13 | 63 | 65 | E var. | ENE | Fine | | |
| 5 | | | 59 | 64 | 53 | 30.00 | 29.85 | 57 | 63 | ESE | ESE | Fine | | |
| 6 | | | 58 | 70 | 53 | 29.80 | 29.74 | 68 | 70 | SE | SE | Fine | | |
| 7 | | | 58 | 75 | 50 | 29.65 | 29.70 | 60 | 75 | SSW | SSW | Fine | | |
| 8 | | | 55 | 65 | 47 | 29.61 | 29.50 | 72 | 81 | SSW | SSW | Fair | Fine | Fair |
| 9 | | .09 | 51 | 60 | 44 | 29.50 | 29.65 | 70 | 72 | SW | SSW | Fair | Fine | Fair |
| 10 | ● | .28 | 49 | 60 | 43 | 29.51 | 29.46 | 75 | 83 | SW | SW | Fine | Fair | Rain |
| 11 | | .13 | 48 | 59 | 39 | 29.50 | 29.42 | 81 | 87 | SW | SW | Fine | Sho'ry | |
| 12 | | | 43 | 62 | 42 | 29.52 | 29.40 | 73 | 75 | W | SSW | Fine | | |
| 13 | | | 47 | 60 | 45 | 29.49 | 29.74 | 74 | 79 | WNW | W var. | Fine | | |
| 14 | | .11 | 49 | 65 | 47 | 30.00 | 29.71 | 71 | 75 | W var. | W var. | Fine | | Sho'ry |
| 15 | | .15 | 49 | 60 | 50 | 30.05 | 29.98 | 68 | 70 | W var. | WSW | Fine | | Sho'ry |
| 16 | | .05 | 54 | 62 | 53 | 29.90 | 29.78 | 73 | 77 | SW | SSW | Sho'ry | Fine | |
| 17 | ☾ | .17 | 58 | 65 | 46 | 29.80 | 29.92 | 79 | 69 | W var. | WNW | Cloud. | Sho'ry | Fine |
| 18 | | | 51 | 60 | 50 | 29.98 | 29.84 | 58 | 71 | SE | ESE | Fine | | |
| 19 | | .03 | 53 | 60 | 52 | 29.67 | 29.56 | 68 | 70 | E | E | Cloud. | | Fair |

The quantity of rain during the month of April, was 1 inch and 90.100ths.

